# Update of the ULtra-early TRranexamic Acid after Subarachnoid Hemorrhage (ULTRA) trial: statistical analysis plan

**DOI:** 10.1186/s13063-020-4118-5

**Published:** 2020-02-18

**Authors:** René Post, Menno R. Germans, Bert A. Coert, Gabriël J. E. Rinkel, W. Peter Vandertop, Dagmar Verbaan

**Affiliations:** 1 Department of Neurosurgery, Neurosurgical Center Amsterdam, Amsterdam University Medical Centers, PO Box 22660, Amsterdam, 1100 DD the Netherlands; 2 Department of Neurosurgery, Clinical Neuroscience Center, University Hospital Zurich, Frauenklinikstrasse 10, 8091 Zurich, Switzerland; 3 Department of Neurology and Neurosurgery, Rudolf Magnus Institute of Neuroscience, University Medical Center Utrecht, PO Box 85060, Utrecht, 3508 AB the Netherlands

**Keywords:** Subarachnoid hemorrhage, Intracranial aneurysm, Tranexamic acid, Clinical outcome, Recurrent bleeding, Statistical analysis plan, ULTRA

## Abstract

**Background:**

Recurrent bleeding from an intracranial aneurysm after subarachnoid hemorrhage (SAH) is associated with unfavorable outcome. Recurrent bleeding before aneurysm occlusion can be performed occurs in up to one in five patients and most often happens within the first 6 h after the primary hemorrhage. Reducing the rate of recurrent bleeding could be a major factor in improving clinical outcome after SAH. Tranexamic acid (TXA) reduces the risk of recurrent bleeding but has thus far not been shown to improve functional outcome, probably because of a higher risk of delayed cerebral ischemia (DCI). To reduce the risk of ultraearly recurrent bleeding, TXA should be administered as soon as possible after diagnosis and before transportation to a tertiary care center. If TXA is administered for a short duration (i.e., < 24 h), it may not increase the risk of DCI. The aim of this paper is to present in detail the statistical analysis plan (SAP) of the ULTRA trial (ULtra-early TRranexamic Acid after Subarachnoid Hemorrhage), which is currently enrolling patients and investigating whether ultraearly and short-term TXA treatment in patients with aneurysmal SAH improves clinical outcome at 6 months.

**Methods/design:**

The ULTRA trial is a multicenter, prospective, randomized, open, blinded endpoint, parallel-group trial currently ongoing at 8 tertiary care centers and 16 of their referral centers in the Netherlands. Participants are randomized to standard care or to receive TXA at a loading dose of 1 g, immediately followed by 1 g every 8 h for a maximum of 24 h, in addition to standard care, as soon as SAH is diagnosed. In the TXA group, TXA administration is stopped immediately prior to treatment (coil or clip) of the causative aneurysm. Primary outcome is the modified Rankin Scale (mRS) score at 6 months after SAH, dichotomized into good (mRS 0–3) and poor (mRS 4–6) outcomes, assessed blind to treatment allocation. Secondary outcomes include case fatalities at 30 days and at 6 months and causes of poor clinical outcome. Safety outcomes are recurrent bleeding, DCI, hydrocephalus, per-procedural complications, and other complications such as infections occurring during hospitalization. Data analyses will be according to this prespecified SAP.

**Trial registration:**

Netherlands Trial Register, NTR3272. Registered on 25 January 2012.

ClinicalTrials.gov, NCT02684812. Registered on 17 February 2016.

## Background

Subarachnoid hemorrhage (SAH) accounts for 5% of all strokes and has an incidence of 7.9 per 100,000 person-years [1]. Only 25% of all patients with aneurysmal SAH have a favorable outcome, and even then, most of these patients still have severe cognitive dysfunction and functional disabilities [2]. The case fatality rate in SAH is approximately 35% due to the initial hemorrhage or subsequent complications. A frequent complication and one of the major causes of death and disability is recurrent bleeding from the aneurysm, which occurs in 4–12% of patients who reach the hospital within the first 24 h [3–9]. The percentage of recurrent bleeding increases to 17% if cases of recurrent bleeding presenting within the first 6 h after the primary hemorrhage (“ultraearly recurrent bleeding”) are also counted [7, 10]. In daily clinical practice, aneurysm treatment is often postponed by either a delay in diagnosis or transfer to a tertiary treatment center [11–13]. Therefore, despite several efforts to improve the logistic processes, ultraearly recurrent bleeding still occurs before the aneurysm is secured. A strategy in addition to early aneurysm occlusion to reduce the number of recurrent bleedings is treatment with antifibrinolytic agents prior to aneurysm occlusion. Results from previous nonrandomized studies using early and short-term administration of antifibrinolytics showed reduction of recurrent bleeding without an increase in delayed cerebral ischemia (DCI) [3, 6, 14, 15]. The only randomized controlled trial of early (< 48 h) and short-term (< 72 h) tranexamic acid (TXA) treatment confirmed a reduction in recurrent bleeding but did not assess the occurrence of DCI and was underpowered to show an effect on clinical outcome [3]. We therefore decided to perform a sufficiently powered randomized clinical trial in which TXA is administrated ultraearly (as soon as possible and at least within the first 24 h after the primary hemorrhage) and for an ultrashort time period (< 24 h) to reduce the risk of the occurrence of DCI. The ULTRA (ULtra-early TRanexamic Acid after subarachnoid hemorrhage) trial is a multicenter, phase III, randomized, controlled, open-label, blinded endpoint trial performed in 8 tertiary care centers and 16 of their referral centers in the Netherlands (see Appendix for list of participants). We published the ULTRA trial protocol previously [16] and now describe the statistical analysis plan (SAP).

### Objectives

The primary aim of the ULTRA trial is to evaluate whether ultraearly and short-term TXA treatment improves clinical outcome after 6 months in patients with SAH.

## Methods/design

### Trial protocol development and conduct

The ULTRA trial is registered with the Netherlands Trial Register (NTR3272; date of registration 25 January 2012) and ClinicalTrials.gov (2012-000343-26; registered on 17 February 2016). The ethics committee of the Amsterdam University Medical Centre (Amsterdam UMC, Amsterdam, the Netherlands) approved the trial protocol on 6 September 2012, starting with two treatment centers and one referral center. Six treatment centers and sixteen referral centers joined the study at a later date. The local accredited ethics committee of each participating hospital approved the local feasibility of the study protocol. During the course of the study, the accredited ethics committee approved three amendments with respect to changes in the inclusion and exclusion criteria. The study was conducted according to the principles of the Declaration of Helsinki, Dutch legislation regarding medical research involving human subjects [17–20], and good clinical practice (GCP) guidelines [21]. Because the majority of patients will not be able to give informed consent at admission, the informed consent procedure for this study is delayed in a so-called emergency procedure as described previously [16]. All study sites were monitored by an independent clinical research associate of the Amsterdam UMC Clinical Research Unit (Amsterdam, the Netherlands). An independent data and safety monitoring board (DSMB) monitored the study’s progress, with a special focus on safety (see below). The trial will be reported according to the Consolidated Standards of reporting Trials (CONSORT) guidelines [22].

Inclusion and exclusion criteria are described in the previously published study protocol [16]. Adult patients with SAH, diagnosed by noncontrast computed tomography (CT) within 24 h after the last hemorrhage, were included. During the trial, “no proficiency of the Dutch or English language” and “treatment for pulmonary embolism” were added to the exclusion criteria, whereas severe liver failure was removed from the exclusion criteria after consultation with the vascular internists. All changes were submitted to the accredited ethics committee as protocol amendments and were approved.

### Randomization and data collection

Patients are randomly allocated in a 1:1 ratio to receive either ultraearly TXA treatment or standard care, stratified by treatment center. TXA is administered as a loading dose of 1 g, immediately followed by 1 g every 8 h for a maximum of 24 h, in addition to standard care, as soon as the SAH is diagnosed. In the TXA group, TXA administration is stopped immediately prior to treatment (coil or clip) of the causative aneurysm. To ensure allocation concealment, the randomization sequence was generated by using GCP-compliant ALEA® randomization software (ALEA Clinical, Abcoude, The Netherlands). Randomization was controlled in each treatment center and web-based, using a dedicated, password-protected, SSL-encrypted website. Data management was implemented according to GCP guidelines. Patients’ data until hospital discharge and 6-month follow-up data are entered via an electronic case record form in a central GCP-compliant web-based database to facilitate on-site data entry (Oracle Clinical®, Redwood Shores, CA, USA; OpenClinica LCC and collaborators, open source software, version 3.6, Waltham, MA, USA, www.OpenClinica.com and Castor Electronic Data Capture, Ciwit BV, Amsterdam, The Netherlands, 2018, www.castoredc.com). Security is guaranteed with login names, login codes, and encrypted data transfer.

#### Primary outcome

The primary outcome is clinical outcome at 6 months measured with the modified Rankin Scale (mRS) score by a standardized and validated telephone interview, performed by a trained research nurse who was blinded to treatment allocation [23, 24]. The mRS is dichotomized into good (mRS 0–3) and poor (mRS 4–6) outcomes [23, 25].

#### Secondary outcomes

Secondary outcomes include mRS score dichotomized into good (mRS scores 0–2) and poor (mRS 3–6) outcomes, ordinal mRS score at 6 months, case fatality at 30 days and at 6 months, causes of poor outcome (directly related to primary SAH, related to a complication of the SAH, related to a complication of treatment, or related to another complication).

#### Safety

Safety outcomes were classified as follows:

1. Complications of SAH (recurrent bleeding, hydrocephalus, DCI)
2. Complications of treatment (per-procedural thromboembolic complication, infarct related to procedure, per-procedural rupture)
3. Other complications (extracranial thrombosis, deep venous thrombosis, pulmonary embolism), hemorrhagic complications, severe hyponatremia, pneumonia, meningitis, urinary tract infection, epilepsy, delirium, and Terson’s syndrome
4. Suspected unexpected serious adverse reactions (SUSARs)
5. Other serious adverse events (SAEs)

Investigators recorded all SAEs during first hospital admission after ictus and reported any adverse event during first hospital admission after ictus that was not related to SAH. Although there are more secondary endpoints, this SAP will focus solely on the clinical (mRS scores and case fatalities) and safety (complications of SAH, complications of treatment, other complications, SUSARs, and other SAEs) secondary endpoints.

### Statistical methods specified in the study protocol

#### Sample size calculation

As described in the study protocol [16], the primary endpoint analysis of this study is based on the difference in percentage of patients with good outcome (mRS score 0 to 3) at 6 months after SAH between patients with and without TXA treatment. It is expected that TXA administration will increase the proportion of patients with a good outcome from 69% to 77.1%.

This expected difference between the TXA and standard care groups was estimated using the results of renowned SAH studies and our own data (293 consecutive patients with aneurysmal SAH, added to angiogram-negative patients with SAH, treated at the AMC between 2008 and 2011). Of all patients with SAH who reach the hospital, 69% have a good outcome (unpublished data). In our data, we find a recurrent bleeding rate of 17%, which is consistent with numbers reported in previous studies (11–22%) [3, 6, 8]. Among patients with recurrent bleeding, an estimated 20% will have a good outcome. Consequently, the percentage of patients with a good outcome without recurrent bleeding is 79%. In the TXA group, the reduction in recurrent bleeding is expected to be 77% [3, 6], which reduces the rate of recurrent bleeding to 3.9%. Furthermore, TXA is anticipated to improve the percentage of good outcome in patients with recurrent bleeding from 20% to 30% [3]. Therefore, in the TXA group, 3.9% will have recurrent bleeding, 30% of whom will have a good outcome. A two-group chi-square test with a 0.05 two-sided significance level will have 80% power to detect the difference between a standard care group proportion of 0.69 and a treatment group proportion of 0.771 (odds ratio, 1.513) when the sample size in each group is 470 (940 patients in total). Taking some withdrawals into account, a total of 950 patients will be included.

#### Originally proposed analyses

In the previously published protocol [16], the originally proposed analyses are described, focusing on the intention-to-treat (ITT) analysis. In the paragraphs below, the final and further detailed SAP is presented, including as-treated (AT) and per-protocol (PP) analyses because use of open-label TXA may modulate possible treatment effects.

#### Interim analysis and safety reporting

A DSMB was installed for this study to protect patients and advise the principal investigator on protecting the safety, validity, and credibility of the trial. Members include a clinically experienced neurologist/epidemiologist, an intensivist, and a statistician. The members were not involved in the trial and had no competing interests. The tasks, responsibilities, and working procedures of the DSMB were described in a charter.

The DSMB performed ongoing safety surveillance (every 6 months), especially with regard to the occurrence of SAEs in terms of increased ischemic events and serious extracranial thrombotic events, such as pulmonary embolism. Every 6 months, the DSMB receives a report prepared by an independent statistician that includes data by treatment group on primary outcome, predefined safety outcomes, other SAEs, and SUSARs. The DSMB also checks the assumptions for sample size calculations without performing statistical analysis. Additionally, the DSMB performed one interim analysis of unblinded effectiveness data during the study. This interim analysis was performed after inclusion of 475 patients in the trial to assess the strength of the efficacy data when half of the patients are enrolled.

The DSMB can recommend the steering committee of the ULTRA trial to:

- Adjust conduct, design, or sample size.
- Terminate the study prematurely when there is clear and substantial evidence of benefit
  - The justifications for a recommendation to terminate the study due to clear benefit will be based on prespecified stopping boundaries for the primary endpoint of the study (mRS score at 6 months). As a stopping rule, the Haybittle-Peto method [26, 27] will be used: interim efficacy analyses (n = 475): *P* = 0.001; final efficacy analyses (*n* = 950): *P* = 0.05.
- Terminate the study prematurely when there is evidence of severe harm
  - The justifications for a recommendation to terminate the study due to clear harm will be based on data showing a notably increase of (serious) adverse events (including case fatalities) in the intervention group. No prespecified formal statistical stopping rule for safety is formulated.
- Terminate the study prematurely in case accrual rates are too low to provide adequate statistical power for identifying the primary endpoint.

If one or more of these situations occurs, the clinical relevance of the results will be incorporated into the decision whether to end the trial prematurely.

### Statistical analysis plan

#### Overall principles

The database will not be unlocked until data regarding efficacy and safety from all patients have been included in the database after data verification and validation are performed and after the SAP has been submitted for publication. The data analysis will start after the 6-month follow-up data of the last included patient have been obtained. Analysis of the primary outcome will be performed according the ITT principle. Given the possible bias of open-label TXA treatment, primary outcome analysis will also be done in an AT population and a PP population to check the robustness of the main analysis, regardless of the presence of statistical significance in the overall analysis. Secondary outcomes will be analyzed in the ITT population, except for the main secondary outcome, mortality at 30 days and at 6 months, which will be analyzed in the ITT, AT, and PP populations. Safety outcomes will be analyzed in the ITT and AT populations. Statistical analyses will be done by the investigators of the ULTRA trial group (see Acknowledgements section). Statistical uncertainty will be expressed in a two-sided 95% confidence interval (CI). Statistical analyses are performed using the IBM SPSS Statistics version 25 software (IBM Corporation, Armonk, NY, USA).

#### Handling of missing data

In case of missing data, every attempt will be made to retrieve the data. Because loss to follow-up is expected to be very low (< 1% missing data on the primary outcome), outcome data will not be imputed. We will state which data are missing and calculate frequencies using the total number of patients with available data. When a patient is lost to follow-up missing his/her 6-month mRS score, this patient cannot be included in the analysis of the primary outcome. If possible, these patients will be included in the secondary outcome analyses. When a patient has withdrawn consent, we will use all available data up until withdrawal of consent [28].

#### Definition of analysis sets

##### ITT population

All randomized patients will be analyzed in the treatment group to which they were originally allocated, regardless of nonadherence or deviations from protocol (Table 5 in Appendix).

##### As-treated population

Patients will be analyzed in groups according to treatment received, regardless of allocated treatment at randomization, thus creating a group that received at least one dose of TXA (intervention) and a group that did not (control). The patients will still be included in the AT analysis if there was a protocol violation (e.g., TXA administration not according to study protocol or not meeting inclusion or exclusion criteria).

##### Per-protocol population

In the PP population, patients allocated to the standard care group who did not receive TXA will be included, as well as patients allocated to the TXA group who received TXA (at least one dose). The patients will still be included in the PP analysis if there was a protocol violation.

### Statistical analyses

#### Patient flow

**Fig. 1:**
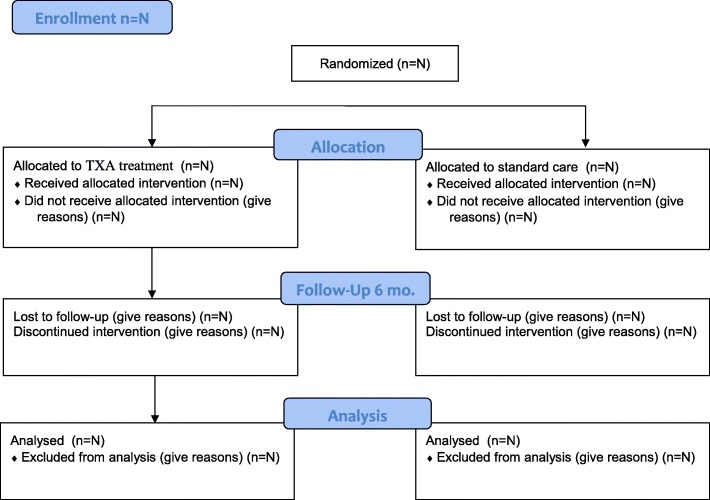
Trial allocation profile (Consolidated Standards of Reporting Trials [CONSORT])

The flow of participants will be displayed in the CONSORT flow diagram (Fig. 1), including the total number of randomized patients and then showing per treatment group the numbers receiving allocated treatment, withdrawing consent, and lost to follow-up.

#### Protocol deviations

When a patient is randomized but does not adhere to inclusion or exclusion criteria, this is considered a protocol deviation regarding eligibility. When a patient is allocated to the standard care group but does receive TXA, or when the patient is allocated to the TXA group but medication administration is not according to the protocol, this is considered a protocol deviation with respect to administration of medication. Protocol deviations will be line-listed in the Appendix.

#### Baseline characteristics

The baseline characteristics of all participants in each treatment group according to allocation will be outlined in a table without formal statistical testing. The table will describe the following variables: age, sex, World Federation of Neurosurgical Societies score, Fisher grade on noncontrast CT on initial (baseline) scan, medication use prior to SAH (antiplatelet therapy, anticoagulants, antihypertensive drugs), location of aneurysm, and treatment modality. Baseline variables will be summarized using simple descriptive statistics (Table 1). Continuous, normally distributed variables will be expressed as means and SDs; continuous, non-normally distributed, and ordinal variables will be expressed as medians (25th–75th percentiles); and categorical variables will be expressed as counts and percentages. Normality of data will be explored by a normal Q-Q plot and tested by the Shapiro-Wilk test.

**Table 1:** Baseline characteristics of participants prior to randomization

	TXA group (*n* = XXX)	Standard care group (*n* = XXX)
Age, yr, mean (SD)	NN.N (NN.N)	NN.N (NN.N)
Female sex, *n* (%)	NNN (X)	NNN (X)
WFNS grade		
I, *n* (%)	N (X)	N (X)
II, *n* (%)	N (X)	N (X)
III, *n* (%)	N (X)	N (X)
IV, *n* (%)	N (X)	N (X)
V, *n* (%)	N (X)	N (X)
Fisher grade		
II, *n* (%)	N (X)	N (X)
III, *n* (%)	N (X)	N (X)
IV, *n* (%)	N (X)	N (X)
Medication prior to SAH		
Platelet inhibitor, *n* (%)	N (X)	N (X)
Anticoagulation, *n* (%)	N (X)	N (X)
Antihypertensive, *n* (%)	N (X)	N (X)
None, *n* (%)	N (X)	N (X)
Location of aneurysm		
Anterior circulation, *n* (%)	N (X)	N (X)
Posterior circulation, *n* (%)	N (X)	N (X)
None, *n* (%)	N (X)	N (X)
Treatment modality		
Endovascular, *n* (%)	N (X)	N (X)
Clipping, *n* (%)	N (X)	N (X)
None, *n* (%)	N (X)	N (X)

*SAH* subarachnoid hemorrhage, *TXA* tranexamic acid, *WFNS* World Federation of Neurosurgical Societies

Data presented as mean (range), *n* (%), or median (IQR), unless noted otherwise

#### Primary outcome

**Fig. 2:**
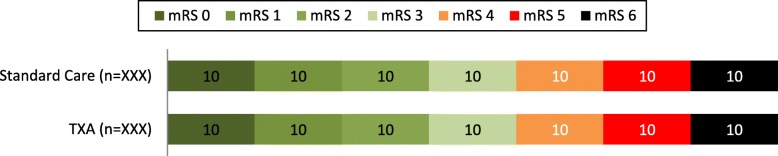
Distribution of mRS at 6 months in the intention to treat analysis

The main statistical analysis will be based on the ITT principle. The occurrence of the primary outcome, dichotomized mRS score at 6 months (good versus poor as mRS 0 to 3 versus mRS 4 to 6, respectively), will be compared between the two treatment groups (Table 2). The distribution of the mRS scores in both treatment groups will be depicted in a histogram (Fig. 2). Treatment effect will be expressed as a difference in proportions with corresponding 95% CI and an odds ratio (OR) estimate with corresponding 95% CI. Additionally, we will analyze the treatment effect on the dichotomized mRS score using multivariable logistic regression, adjusting for the stratification variable (treatment center) and, if necessary, clinically relevant baseline imbalances. Effect size will be expressed as an adjusted OR. The crude and adjusted analyses will also be performed in both the AT and PP populations.

**Table 2:** Primary outcome (modified Rankin Scale score at 6 months) and secondary outcomes

	ITT			
	TXA group	Standard care group	OR (95% CI)	aOR (95% CI)
mRS 0–3	XX	XX	XX (XX–XX)	XX (XX–XX)
Mortality at 30 days	XX	XX	XX (XX–XX)	XX (XX–XX)
Mortality at 6 mo	XX	XX	XX (XX–XX)	XX (XX–XX)

*aOR* adjusted odds ratio, *CI* confidence interval, *ITT* intention to treat, *mRS* modified Rankin Scale, *OR* odds ratio, *TXA* tranexamic acid

#### Sensitivity analyses

Dichotomized mRS score is chosen as the primary outcome because results of the analysis are straightforward and easy to interpret. However, it is also clear that the cutoff is arbitrarily chosen and information is lost by dichotomization. Ordinal analysis of outcome data is becoming increasingly more common in acute stroke trials because it increases statistical power [29]:

Sensitivity analyses play a crucial role in assessing the robustness of the findings or conclusions based on primary analyses of data in clinical trials. They are a critical way to assess the impact, effect or influence of key assumptions or variations—such as different methods of analysis, definitions of outcomes, protocol deviations, missing data, and outliers—on the overall conclusions of a study [30].

Therefore, two sensitivity analyses will be performed: first, the dichotomized mRS using the cutoff frequently used in stroke (good outcome, mRS scores 0–2) will be analyzed using the same analysis as described by the primary outcome; second, the ordinal mRS score will be analyzed using an ordinal regression model on the total range of the mRS under the assumption of proportional odds (Table 3). If the assumption of ordinal regression does not hold, we will perform sliding dichotomy analysis [31].

**Table 3:** Sensitivity analysis

	ITT			
	TXA group	Standard care group	OR (95% CI)	aOR (95% CI)
Excellent outcome (mRS 0–2)	NN (X %)	NN (X %)	X.XX (X.XX–X.XX)	X.XX (X.XX–X.XX)
Ordinal shift mRS)	NN (X %)	NN (X %)	X.XX (X.XX–X.XX)	X.XX (X.XX–X.XX)
mRS0				
mRS1				
mRS2				
mRS3				
mRS4				
mRS5				
mRS6				

Data are *n* (%), mean (SD), or median (IQR)

*aOR* adjusted odds ratio, *CI* confidence interval, *ITT* intention to treat, *mRS* modified Rankin Scale, *OR* odds ratio, *TXA* tranexamic acid

When the loss to follow-up rate is > 10%, a third sensitivity analysis will be performed. Data will be analyzed according to a worst case scenario; that is, patients lost to follow-up in the treatment group will have the worst possible outcome, and patients in the standard care group will have the best possible outcome.

#### Secondary outcomes

The secondary outcome analyses will compare case fatality at 30 days and at 6 months, causes of death or poor outcome at 6 months, and all safety outcomes between treatment groups (Table 4). The statistical analysis will also be based on the ITT principle. Treatment effect will be expressed in a difference in proportions with corresponding 95% CI and an odds ratio (OR) estimate with corresponding 95% CI. The analyses for the main secondary outcome, mortality at discharge and at 6 months, will also be performed in both the AT and PP populations. The analyses for the safety outcomes will also be performed in the AT population.

**Table 4:** Safety outcomes during hospital admission

	ITT		
	TXA group (*n* = xx)	Standard care group (*n* = xx)	OR (95% CI)
Any SAE, *n* (%)	NN (X)	NN (X)	X.XX (X.XX–X.XX)
Recurrent bleeding	NN (X)	NN (X)	X.XX (X.XX–X.XX)
Hydrocephalus	NN (X)	NN (X)	X.XX (X.XX–X.XX)
Delayed cerebral ischemia	NN (X)	NN (X)	X.XX (X.XX–X.XX)
Thromboembolic complications during treatment Coiling, *n* (%)	NN (X)	NN (X)	X.XX (X.XX–X.XX)
Infarct related to procedure Clipping, *n* (%)	NN (X)	NN (X)	X.XX (X.XX–X.XX)
Procedural rupture Coiling, *n* (%) Clipping, *n* (%)	NN (X)	NN (X)	X.XX (X.XX–X.XX)
Extracranial thrombosis	NN (X)	NN (X)	X.XX (X.XX–X.XX)
- DVT	NN (X)	NN (X)	X.XX (X.XX–X.XX)
- PE	NN (X)	NN (X)	X.XX (X.XX–X.XX)
Hemorrhagic complication	NN (X)	NN (X)	X.XX (X.XX–X.XX)
Severe hyponatremia	NN (X)	NN (X)	X.XX (X.XX–X.XX)
Pneumonia	NN (X)	NN (X)	X.XX (X.XX–X.XX)
Meningitis	NN (X)	NN (X)	X.XX (X.XX–X.XX)
Urinary tract infection	NN (X)	NN (X)	X.XX (X.XX–X.XX)
Epilepsy	NN (X)	NN (X)	X.XX (X.XX–X.XX)
Delirium	NN (X)	NN (X)	X.XX (X.XX–X.XX)
Terson’s syndrome	NN (X)	NN (X)	X.XX (X.XX–X.XX)
SUSARs	NN (X)	NN (X)	X.XX (X.XX–X.XX)
Other	NN (X)	NN (X)	X.XX (X.XX–X.XX)

*CI* confidence interval, *DVT* deep venous thrombosis, *ITT* intention to treat, *OR* odds ratio, *PE* pulmonary embolism, *SAE* serious adverse event, *SUSARs* suspected unexpected serious adverse reactions, *TXA* tranexamic acid

### Trial status

Initially, two treatment centers started recruitment between July 2013 and February 2014, and six additional treatment centers started recruitment between April 2014 and September 2016. A total of 16 referral centers started recruitment between July 2013 and November 2018. All participating centers are in the Netherlands. Currently, we have enrolled all 955 patients.

## Data Availability

The datasets used and/or analyzed during the current study are available from the corresponding author on reasonable request.

## Appendix

**Table 5:** Definition of population analysis sets

Analysis population	TXA group	Standard care group
Intention to treat (“as randomized”)	Patients randomized to TXA group: • Including all protocol deviations	Patients randomized to standard care group: • Including all protocol deviations
As treated (“actual treatment”)	Patients who received TXA (at least one dose), regardless of allocated treatment at randomization: • Including patients who received TXA as described according to protocol • Including patients who received TXA not following protocol (protocol deviation) • Including patients with other protocol deviations	Patients who did not receive TXA, regardless of allocated treatment at randomization: • Including patients who received standard care as described according to protocol • Including patients who received standard care not following protocol (protocol deviation) • Including patients with other protocol deviations
Per protocol	Patients randomized to TXA group who received TXA (at least one dose): • Including patients who received TXA as described according to protocol • Including patients who received TXA not following protocol (protocol deviation) • Including patients with other protocol deviations	Patients randomized to standard care group who did not receive TXA: • Including patients who received standard care as described according to protocol • Including patients who received standard care not following protocol (protocol deviation) • Including patients with other protocol deviations

*TXA* tranexamic acid

## Abbreviations

aOR: Adjusted odds ratio
AT: As treated
CI: Confidence interval
CONSORT: Consolidated Standards of Reporting Trials
CT: Computed tomography
DCI: Delayed cerebral ischemia
DSMB: Data and safety monitoring board
DVT: Deep venous thrombosis
GCP: Good clinical practice
ITT: Intention to treat
mRS: Modified Rankin Scale
OR: Odds ratio
PE: Pulmonary embolism
PP: Per protocol
SAE: Serious adverse event
SAH: Subarachnoid hemorrhage
SAP: Statistical analysis plan
SUSAR: Suspected unexpected serious adverse reaction
TXA: Tranexamic acid
WFNS: World Federation of Neurosurgical Societies
